# Interactions of the periplasmic binding protein CeuE with Fe(III) n-LICAM^4−^ siderophore analogues of varied linker length

**DOI:** 10.1038/srep45941

**Published:** 2017-04-06

**Authors:** Ellis J. Wilde, Adam Hughes, Elena V. Blagova, Olga V. Moroz, Ross P. Thomas, Johan P. Turkenburg, Daniel J. Raines, Anne-Kathrin Duhme-Klair, Keith S. Wilson

**Affiliations:** 1 Structural Biology Laboratory, Department of Chemistry, University of York, Heslington, York YO10 5DD, UK; 2 Department of Chemistry, University of York, Heslington, York YO10 5DD, UK

## Abstract

Bacteria use siderophores to mediate the transport of essential Fe(III) into the cell. In *Campylobacter jejuni* the periplasmic binding protein CeuE, an integral part of the Fe(III) transport system, has adapted to bind tetradentate siderophores using a His and a Tyr side chain to complete the Fe(III) coordination. A series of tetradentate siderophore mimics was synthesized in which the length of the linker between the two iron-binding catecholamide units was increased from four carbon atoms (4-LICAM^4−^) to five, six and eight (5-, 6-, 8-LICAM^4−^, respectively). Co-crystal structures with CeuE showed that the inter-planar angles between the iron-binding catecholamide units in the 5-, 6- and 8-LICAM^4−^ structures are very similar (111°, 110° and 110°) and allow for an optimum fit into the binding pocket of CeuE, the inter-planar angle in the structure of 4-LICAM^4−^ is significantly smaller (97°) due to restrictions imposed by the shorter linker. Accordingly, the protein-binding affinity was found to be slightly higher for 5- compared to 4-LICAM^4−^ but decreases for 6- and 8-LICAM^4−^. The optimum linker length of five matches that present in natural siderophores such as enterobactin and azotochelin. Site-directed mutagenesis was used to investigate the relative importance of the Fe(III)-coordinating residues H227 and Y288.

The periplasmic binding protein (PBP) CeuE^1^ is an important component of the Fe(III) uptake system in *Campylobacter jejuni*, a commensal Gram-negative bacterium in the avian gut, but a common source of food poisoning in humans. The acquisition of iron poses a severe challenge for many bacteria due to the instability of Fe(II) in aqueous aerobic environments coupled with the very low aqueous solubility of Fe(III) – around 10^−17^ M. In addition, eukaryotic hosts use their own Fe(III) sequestering systems to limit the extracellular iron concentration to 10^−24^ M^2^,^3^,^4^. As bacteria typically require a cellular iron concentration of 10^−6^ to 10^−8^ M for optimal growth, powerful bacterial iron uptake systems have evolved, several of which involve the biosynthesis and secretion of siderophores, small molecule iron chelators that are some of the strongest Fe(III)-binding compounds known^4^,^5^,^6^,^7^. Over 500 different siderophores have now been identified and have been widely studied and reviewed^2^,^5^,^6^,^8^,^9^,^10^,^11^. Siderophores can be categorized by the chemical moieties involved in iron coordination, with four main classes: catecholates, hydroxamates, α-hydroxy-carboxylates, or mixed-type, (Fig. 1)^5^. Many contain a hexadentate arrangement of hard donor atoms to complement the binding requirements of Fe(III), a hard Lewis acid, although some have fewer than six chelating atoms.

Enterobactin, a well-characterized hexadentate catecholate siderophore (Fig. 1), is one of the strongest Fe(III) chelators known at physiological pH, and is synthesized and secreted by a number of species including *Escherichia coli*^2^. While enterobactin and its derivatives have a backbone linker length of five atoms between the catecholamide groups, there is some variation in linker length in other naturally occurring siderophores. For example, bacillibactin, a hexadentate catecholate siderophore, has an extended backbone of 11 atoms^12^ while the amonabactins, synthesized by *Aeromonas hydrophila*, form a tetradentate biscatecholate siderophore family containing tryptophan or phenylalanine side chains, with a Lys-Lys 12 atom or even longer Lys-Lys-Gly 15 atom linker (Fig. 1)^13^,^14^,^15^.

In Gram-negative bacteria, Fe(III)-siderophore complexes are transported into the periplasm, where they are captured by specific PBPs^16^. The PBP then delivers the Fe(III)-siderophore complexes to a transporter in the inner membrane for delivery into the cytoplasm, where the Fe(III) is released^6^. The crystal structures of a number of siderophore PBPs have been determined, and while their sequence identity can be as low as 15%, they are structurally similar, being comprised of two domains connected through a long α-helical backbone^16^.

*C. jejuni* does not itself synthesize siderophores, but capitalizes on their secretion by other bacteria, such as *E. coli*. CeuE, an integral part of the CeuBCDE ABC transporter of *C. jejuni*, is responsible for capturing Fe(III)-siderophore complexes in the periplasm for delivery to their respective inner membrane transporter. It is not surprising that, as a scavenger, *C. jejuni* can utilize a diverse range of catecholate siderophores for the uptake of Fe(III), including tetradentate siderophores such as azotochelin and the enterobactin-derived bis(2,3-dihydroxybenzoyl-L-Ser) (Fig. 1)^17^. Indeed, the periplasmic enterobactin esterase Cee has been shown to play an important role in the uptake of Fe(III) in *C. jejuni*, since it hydrolyzes the enterobactin backbone in the periplasm followed by the import of Fe(III)-enterobactin fragment complexes, including bis(2,3-dihydroxybenzoyl-L-Ser), into the cytoplasm^18^. The observation that there is no known cytoplasmic enterobactin esterase in *C. jejuni* suggests that hexadentate analogues may first require cleavage to their tetradentate counterparts before transportation into the cytoplasm^18^.

Previously we cloned and overexpressed CeuE^1^, synthesized two catecholate siderophore analogues and probed their interaction with the protein using a combination of solution and structural approaches^19^,^20^. The first complex was with MECAM^6−^ (**PDB ID: 2CHU**)^19^, a synthetic enterobactin analogue that uses three catecholate arms to chelate Fe(III). However, since studies showed that tetradentate bis-catecholates can also act as siderophores for other bacterial species^21^, we subsequently synthesized the tetradentate analogue 4-LICAM^4−^, which contains a four-atom linker between the two catecholamide units (Fig. 1). Co-crystallization of its Fe(III) complex with CeuE revealed the formation of a 1:1:1 complex CeuE-[Fe^III^(4-LICAM)]^−^ (**PDB ID: 5A1J**^20^). The octahedral coordination of the Fe(III) ion was completed by two protein residues, Tyr288 and His227. Subsequent studies^22^ showed that 4-LICAM^4−^ was a good mimic of the natural tetradentate linear enterobactin hydrolysis product: bis(2,3-dihydroxybenzoyl-L-Ser) (**PDB ID: 5ADV, 5ADW**), (Fig. 1). Four homologues of CeuE identified using the Dali server (**PDB ID: 3GFV, 4JCC, 3TEF and 4MX8**) with Z-scores greater than 30, were all shown to contain the conserved Tyr and His residues, suggesting that this mode of binding is conserved in these bacteria^23^.

Here we report a combined solution and structural study of a series of synthetic tetradentate analogues of the enterobactin-derived bis(2, 3-dihydroxybenzoyl-L-Ser), to explore the ligand-binding promiscuity of CeuE, both wild type and with mutations of the Fe(III) binding residues H227 and Y288. The length of the linker between the Fe(III)-binding catecholamide units was increased from four carbon atoms (4-LICAM^4−^) to five, six and eight (5-, 6-, 8-LICAM^4−^, respectively). Site directed mutagenesis was used to probe the relative contribution of H227 and Y288 to ligand binding.

Fe(III) complexes of 5-LICAM^4−^, 6-LICAM^4−^ and 8-LICAM^4−^ were co-crystallized with CeuE and their crystal structures determined. The geometrical parameters obtained and those previously published were then correlated with the dissociation constants of the four synthetic [Fe^III^(n-LICAM)]^−^ complexes, with n = 4, 5, 6 or 8, from CeuE, as measured by intrinsic fluorescence quenching. Analogous studies were carried out with CeuE mutants in which His227 and Tyr288 were replaced with non-coordinating residues.

## Results and Discussion

The binding of all the n-LICAM ligands was first measured for wild type CeuE. To probe the relative contributions of His227 and Tyr288 to Fe(III)-siderophore binding, site-directed mutagenesis was then used to replace the side chains of the two Fe(III)-coordinating amino acids with non-coordinating side chains of similar size. His227 was mutated to either leucine (H227L) or alanine (H227A), while Y288 was mutated to phenylalanine (Y288F). In addition, the double mutants H227L/Y288F and H227A/Y288F were created.

### Crystal structures of the wild type ligand complexes

#### Overall fold

**Figure 2:**
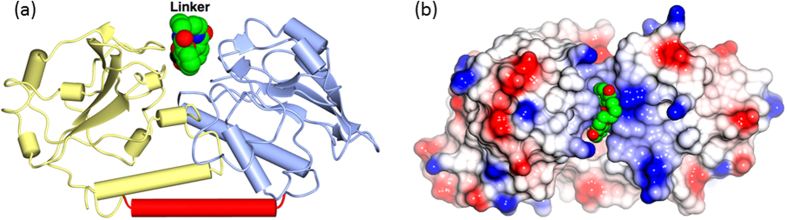
(**a**) The fold of CeuE in the CFe-5-Lic complex shown as worm and tubes. The N-terminal domain (ice blue, residues 22–159) is linked to the C-terminal (yellow, residues 178–310) by a long α-helix (red). The ligand is shown as van der Waals spheres and lies at the interface of the two domains. This and the other structural figures were made using CCP4 mg^25^. (**b**) The CFe-5-Lic binding site viewed from the solvent, with the protein shown as an electrostatic surface and the Fe-n-Lic as van der Waals spheres. The five carbon atoms of the linker lie on the surface of the complex, with the two catecholate groups pointing down into pockets where they chelate the iron, burying it in the cleft.

For brevity, the four [Fe^III^(n-LICAM)]^−^ complexes will henceforth be referred to as Fe-4-Lic, Fe-5-Lic, Fe-6-Lic or Fe-8-Lic and the four CeuE-[Fe^III^(n-LICAM)]^−^ complexes as CFe-4-Lic, CFe-5-Lic, CFe-6-Lic or CFe-8-Lic. The overall fold of *C. jejuni* CeuE has been described in detail previously^19^,^20^, and the structure of CFe-4-Lic has also been reported^19^,^20^. The ligand lies in a cleft between the two domains of the protein, shown for CFe-5-Lic in Fig. 2a and b. In the three co-crystal structures, CFe-5-Lic, CFe-6-Lic and CFe-8-Lic, there is well defined density for the whole protein chain and the overall fold is very similar to that in CFe-4-Lic (**PDB ID: 5A1J**), with an r.m.s.d. of 0.38 Å (288 Cα positions), 0.48 Å (287 Cα positions) and 0.45 Å (288 Cα positions) calculated using the SSM algorithm^24^. There is very little change in the structure of the protein upon ligand binding, as is shown by superposing all co-crystal structures on Chain A of apo-CeuE (the three independent chains in the apo protein are essentially identical) giving r.m.s.d.s of 0.90 (285 Cα positions) Å, 0.94 Å (283 Cα positions) and 0.89 Å (285 Cα positions).

#### The siderophore-binding pocket

**Figure 3:**
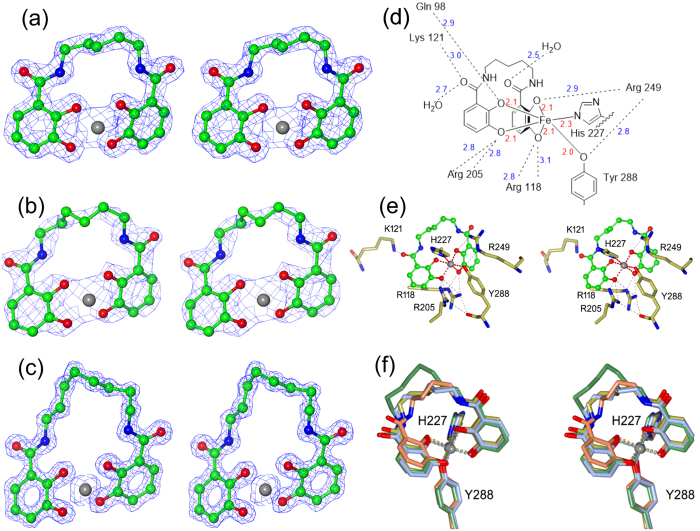
Close up view of (**a**) CFe-5-Lic, (**b**) CFe-6-Lic and (**c**) CFe-8-Lic with the electron density for the maximum likelihood weighted map contoured at the 2σ level. n-Lics are shown as ball and stick colored by atom type, the Fe(III) cation as a gray sphere. (**d**) Schematic diagram of all protein-ligand contacts when Fe-5-Lic is bound in the CeuE binding pocket. Fe-donor atom bond distances (Å) are shown in red. All other bind distances (Å) are shown in blue. (**e**) Close up view of the ligand-binding site. 5-Lic is shown as ball and stick colored by atom type, the Fe(III) cation as a grey sphere with its contacts in red and the neighboring side chains as cylinders with the carbon atoms in gold. H-bonds are shown as dotted lines. (**f**) Close-up of the CFe-4-Lic (coral) CFe-5-Lic, (gold) CFe-6-Lic (ice blue) and CFe-8-Lic (green) binding sites and coordinating residues after superposition using SSM^24^.

There is well-defined electron density for the Fe(III) cation and the n-Lic ligand in all three complexes, as shown in Fig. 3(a–c). The linkers between the two catecholate binding units are well ordered, with B-values close to the average value for each structure, in spite of the linker pointing toward the solvent with essentially no contacts to the protein (Fig. 2). The longer linker of 8-Lic is notably well ordered considering its length and inherent potential for flexibility. This tolerance of longer linkers can be rationalized by the lack of protein contacts observed for the linker region in the structures. As in the CFe-4-Lic structure, in the three new complexes the octahedral coordination sphere of the iron centers is composed of four oxygen donors from the two catecholate groups with the two remaining donor atoms being provided by the side chains of His227 and Tyr288, Fig. 3(d–f)^20^. The residues that contribute to the siderophore complex binding are depicted in Fig. 3(e) for CFe-5-Lic. Three arginine residues, Arg118, Arg205 and Arg249, interact with the catechol oxygen atoms via hydrogen bonding. In addition, there are hydrogen bonds between Lys121 and a catechol oxygen, and Gln98 and an amide carbonyl. Other than Gln98, only water is involved in hydrogen bonding to the linker region.

**Figure 4:**
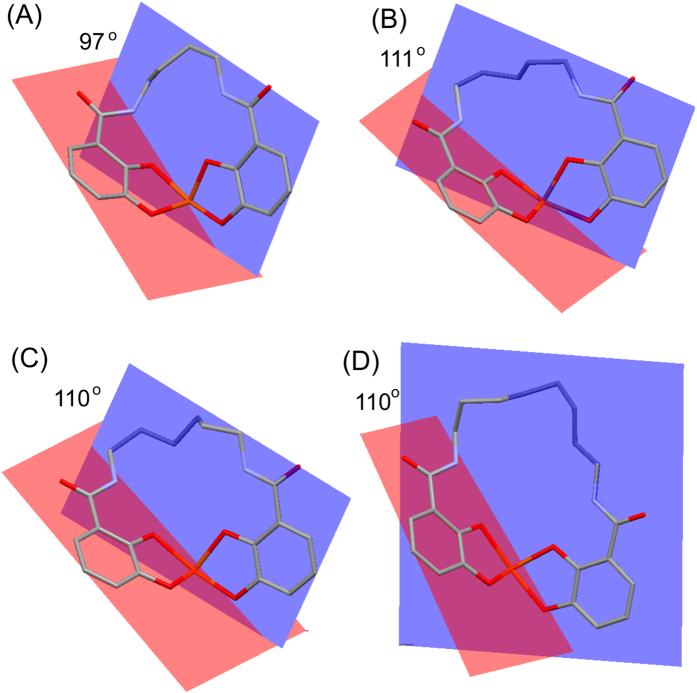
Inter-planar angles between mean planes defined by the aromatic carbon atoms in (**a**) CFe-4-Lic, (**b**) CFe-5-Lic, (**c**) CFe-6-Lic and (**d**) CFe-8-Lic (images produced using Mercury 3.5.1).

Superposition of the four structures, Fig. 3(f), reveals subtle differences in the binding mode. The accommodation of the four, five, six or eight carbons in the linker is seen at the top of Fig. 3(f). The catecholate unit shown on the right hand side has an almost identical location in all four complexes. In contrast, the catecholate unit shown on the left, in essentially the same position in 5-, 6- and 8-Lic, is pulled upwards by the shorter linker in 4-Lic; nevertheless, its two iron-chelating oxygen atoms remain in positions very close to those seen in the other three structures. The structural differences can be quantified by calculating the mean planes defined by the six carbon atoms of the two catecholate rings, and measuring their inter-planar angle which is 97°, 111°, 110° and 110° in CFe-4-Lic, CFe-5-Lic, CFe-6-Lic and CFe-8-Lic, respectively (Fig. 4). It is evident that Fe-4-Lic cannot bind with the optimum inter-planar angle of ~110° due to its short butylene linker. In contrast, the longer and more flexible linkers in Fe-5-Lic, Fe-6-Lic and Fe-8-Lic can adapt to both the geometric requirements of the Fe(III) center and the binding pocket of CeuE.

### Crystal structures of the mutants

**Figure 5:**
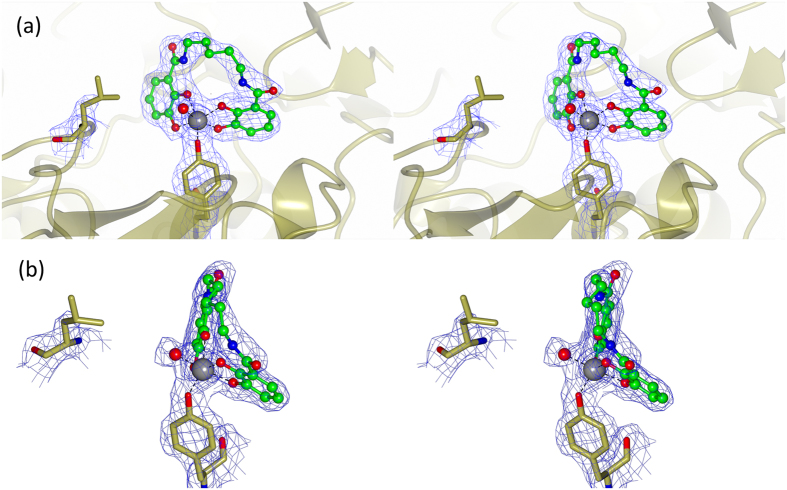
(**a**) Crystal structure of H227L-Fe-5-Lic with the electron density for the maximum likelihood weighted map contoured at the 2σ level. The Y288 and L227 sidechains are shown in cylinders with carbon atoms in gold. Fe(III) atom is shown as a gray sphere, and the 5-Lic ligand in ball and stick colored by atom type. L227 is located away from the binding pocket, with a water molecule (red sphere) completing the octahedral coordination sphere. (**b**) Alternate view of crystal structure of H227L-Fe-5-Lic showing hexacoordinate Fe(III) with a water ligand.

All five mutants (Y288F, H227L, H227A, H227L/Y288F, H227A/Y288F) were crystallized in their apo form, and all structures are closely similar to that of wild type CeuE. All mutations were confirmed by sequencing of the DNA. However while there is clear electron density for the Y288F mutation in its three structures, H227L and H227A are located in a flexible loop region and there is generally no electron density to confirm their presence. Co-crystallization of mutant H227L with Fe-5-Lic yielded a structure containing three protein ligand complexes in the asymmetric unit (**PDB ID: 5TCY**). The loop region upon which the H227L mutation is ordered, but points away from the Fe(III) coordination sphere. A water molecule completes the octahedral Fe(III) coordination (with good density in only one of the three independent molecules in the asymmetric unit, Fig. 5).

### Intrinsic fluorescence quenching

**Figure 6:**
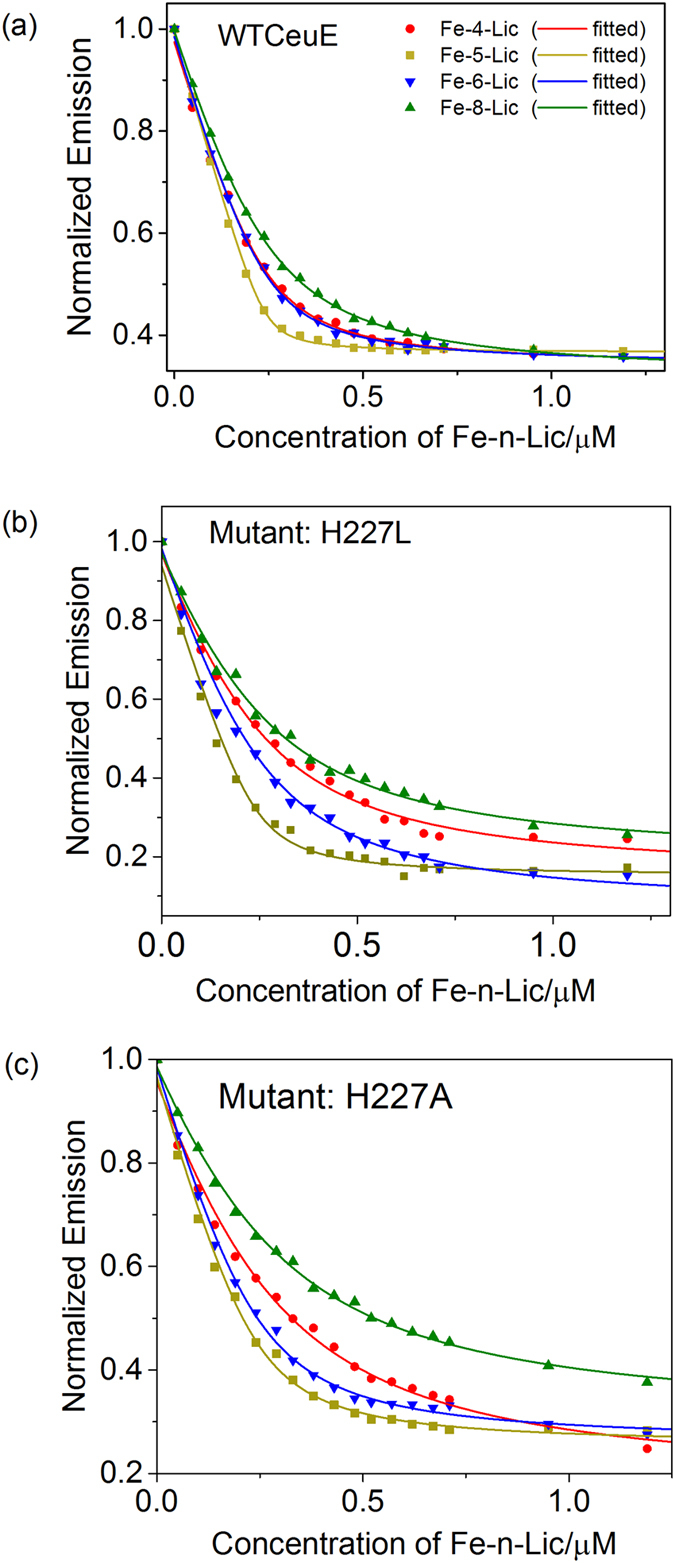
Selected fluorescence quenching data for binding to (**a**) CeuE (**b**) mutant H227L and (**c**) mutant H227A. Fe-4-Lic (red circles), Fe-5-Lic (gold squares), Fe-6-Lic (blue inverted triangles) and Fe-4-Lic (green triangles) (240 nM protein in 40 mM TrisHCl pH 7.5, NaCl 150 mM).

The affinities of CeuE for the four Fe-n-Lic complexes were quantified using intrinsic fluorescence quenching, Table 1, with measurements carried out in triplicate. The data were fitted to a 1:1 binding model and analyzed by non-linear regression^26^. Whilst the binding of Fe-5-Lic was too tight to allow the calculation of an accurate dissociation constant, a comparison of the shape of the individual titration curves suggest that Fe-5-Lic binds slightly more tightly than either Fe-4-Lic or Fe-6-Lic (Fig. 6(a)). An attempt to determine the *K*_d_ value of Fe-5-Lic by using a more dilute protein solution was unsuccessful because of the inherent decrease in emission intensity reaching the detection limit of the fluorimeter.

The *K*_d_ values of Fe-4-Lic and Fe-6-Lic are similar and lie within three standard deviations of one another. This indicates that the entropic cost incurred upon binding of the ligand with the more flexible 6-atom linker is compensated by its ability to accommodate the preferred octahedral coordination geometry of Fe(III). Since the inter-planar angle between the catecholamide units in the CFe-6-Lic and CFe-8-Lic structures is identical (110°), the higher *K*_d_ value obtained for Fe-8-Lic is likely to reflect the higher entropic cost upon binding due to the greater number of conformational degrees of freedom in its linker. Overall, the relative similarity of the dissociation constants for the series shows that the linker length has only a subtle effect on binding affinity to CeuE.

**Figure 7:**
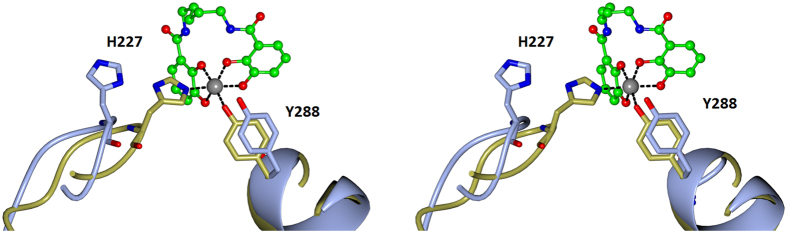
Overlay of the apo structure of CeuE (ice blue) (PDB ID: 3ZKW) with that of CFe -5-Lic (gold) (PDB ID: 5A5D). Tyr288, located on a helical region, has only to shift slightly to coordinate, whilst His227, located on a flexible loop, has to move significantly to reach the Fe(III) center.

The binding studies with wild type CeuE were followed by titrations to determine the ligand affinity of all five mutants. Fe-5-Lic was chosen because of its strong binding to wild type CeuE and because its linker length matches that of natural catecholate siderophores. Mutation of the Fe(III)-coordinating tyrosine led to a drastic drop in binding affinity in both the single (Y228F) and the double mutants (H227L/Y288F and H227A/Y288F), with *K*_d_ values above 1 μM. In contrast, the effect of mutating the histidine was less pronounced. Mutants H227L and H227A gave *K*_d_ values of 22 ± 10 nM and 35 ± 14 nM, respectively, indicating only slightly weaker binding than for wild type, which indicates that the tyrosinate O-donor binds the Fe(III) cation more strongly than the histidine N-donor. However, there is an additional entropic contribution to consider. Comparison of the apo structure of CeuE (**PDB ID: 3ZKW**) with that of CFe-5-Lic (**PDB ID: 5A5D**) shows that upon ligand binding, Tyr288 only has to shift slightly to coordinate, whilst His227, located on a flexible loop, has to move significantly to reach the Fe(III) center (Fig. 7). Hence, the coordination of the latter will have an entropic cost associated with it.

Subsequently, titrations were carried out with Fe-4-Lic, Fe-6-Lic and Fe-8-Lic to identify trends in the affinity for mutants H227L and H227A (Fig. 6(b) and (c)) (Table 1). The resulting K_d_ values show trends similar to that observed for wild type, but with systematically weaker binding.

### CD spectroscopy

**Figure 8:**
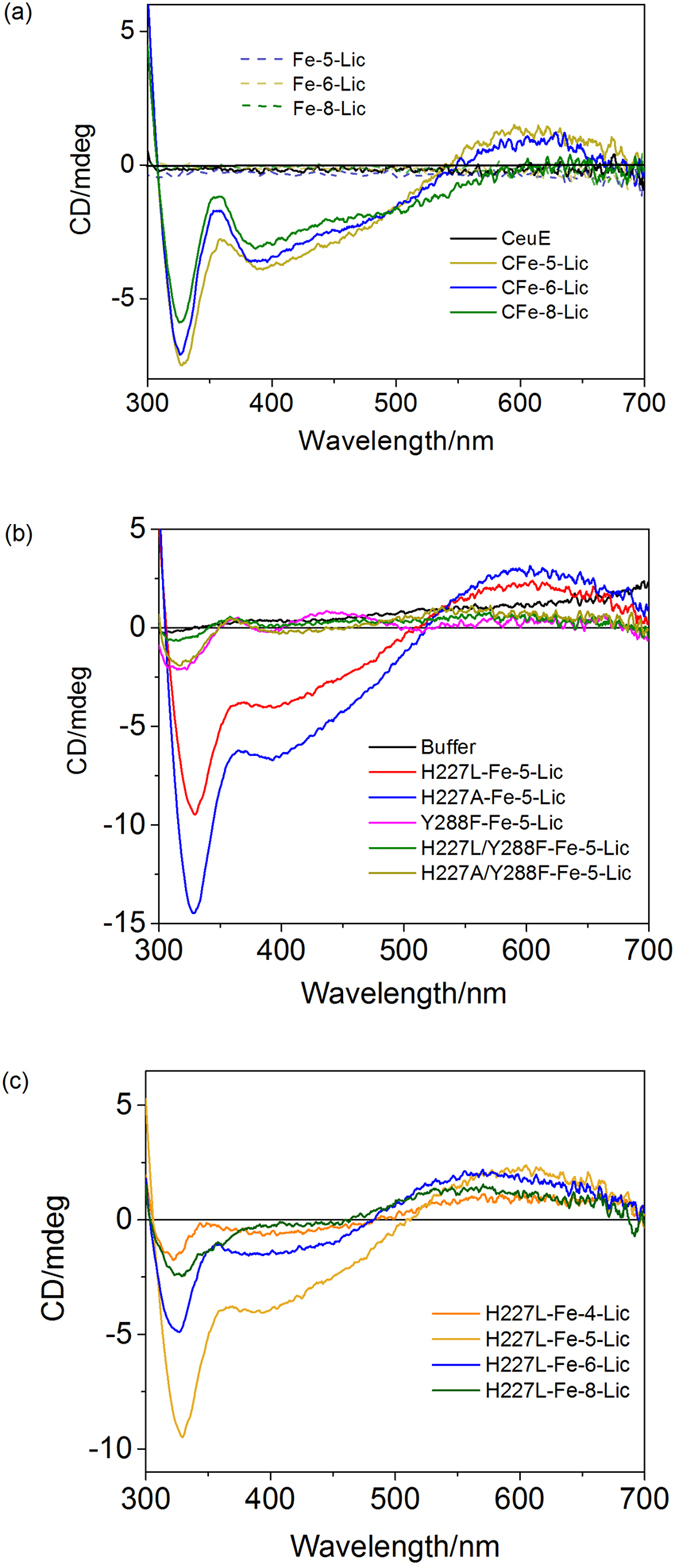
(**a**) CD spectra of CFe-5-Lic (gold), CFe-6-Lic (blue), CFe-8-Lic (green), Fe-5-Lic (dotted gold), Fe-6-Lic (dotted blue) and Fe-8-Lic (dotted green) in the absence of CeuE and CeuE in the absence of Fe(III) and n-Lic (black); concentrations 50 μM in 100 mM TRIS buffer, 150 mM NaCl, 0.5% DMSO, pH 7.5. (**b**) CD spectra of Fe-5-Lic in the presence of all mutant proteins: H227L-Fe-5-Lic (red), H227A-Fe-5-Lic (blue), Y288F-Fe-5-Lic (pink), H227L/Y288F-Fe-5-Lic (green), H227A/Y288F-Fe-5-Lic (gold); concentrations 50 μM in 100 mM TRIS buffer, 150 mM NaCl, 0.5% DMSO, pH 7.5. (**c**) CD spectra of H227L-Fe-4-Lic (coral), H227L-Fe-5-Lic (gold), H227L-Fe-6-Lic (blue), H227L-Fe-8-Lic (green), concentrations 50 μM in 100 mM TRIS buffer, 150 mM NaCl, 0.5% DMSO, pH 7.5.

The crystal structures of all three complexes show that the siderophore analogues are bound in the Λ-configuration, as previously reported for CFe-4-Lic^20^, (CeuE)_2_-[(Fe(MECAM))_2_]^6− ^19, FeuA-[Fe(MECAM)]^3−^, FeuA-[Fe(enterobactin)]^3−^ and FeuA-[Fe(bacillibactin)]^3^,^27^,^28^. To confirm that the preference for the Λ-configuration is retained in solution, circular dichroism (CD) spectroscopy was used to probe the ligand-to-metal charge transfer (LMCT) band (Fig. 8(a)). The minimum and maximum of the region of the LMCT band at approximately 408 nm and 595 nm, respectively, are indicative of the Λ-configuration^29^, with no CD signal observed in the absence of either the protein or ligand, confirming that the protein selectively binds the Λ-configuration. The decrease in amplitude of the signal with the increase in the number of spacer atoms indicates that CeuE binds the ligands in the order Λ-Fe-5-Lic > Λ-Fe-6-Lic > Λ-Fe-8-Lic, in agreement with the dissociation constants recorded by fluorescence titration.

Circular dichroism spectroscopy was used to investigate if the mutants also induced Λ-configuration in Fe-5-Lic. The Y228F mutants gave no signal, confirming a lack of Λ-configuration induction, which may indicate a lack of ligand binding. The Λ-configuration signal was observed for mutants H227L and H227A, indicating that these do indeed bind Λ-Fe-5-Lic (Fig. 8(b)). Complexes of H227L and H227A were then prepared with all four Fe-n-Lic complexes. The Λ-configuration was observed for all four, but with a decrease in signal amplitude compared to wild type (Fig. 8(c)), confirming the reduced binding affinity observed in the fluorescence quenching experiments.

## Summary and Conclusions

The tetradentate siderophore mimics Fe-5-Lic, Fe-6-Lic and Fe-8-Lic were co-crystallized with the periplasmic binding protein CeuE of *C. jejuni.* In addition to electrostatic interactions and hydrogen bonding, the interactions of the binding pocket of CeuE with all three siderophore complexes involved the direct co-ordination of two amino acid side chains (His227 and Tyr288) to the Fe(III) center, as previously observed for CFe-4-Lic. The inter-planar angle between the Fe(III)-coordinated catecholamide units increases from a strained 97° found in CFe-4-Lic to 111°, 110° and 110° in CFe-5-Lic, CFe-6-Lic and CFe-6-Lic, respectively. As in our previously determined co-crystal structures, CeuE was found to select for Λ-configured Fe(III) complexes in all these structures.

In addition, the affinities of CeuE for Fe-4-Lic, Fe-5-Lic, Fe-6-Lic and Fe-8-Lic were determined using fluorescence quenching, which yielded dissociation constants of 21 ± 6 nM, < 10 nM, 33 ± 8 nM and 58 ± 8 nM, respectively. These results indicate that CeuE exhibits a degree of ligand-binding promiscuity and that the length of the linker between the Fe(III)-binding catecholamide units has a subtle effect on the binding of the bis(catecholate) siderophores, which is driven by the balance between the enthalpic gain through optimized interactions with the binding pocket *vs* entropic costs associated with the loss of conformational degrees of freedom. Accordingly, the binding affinity initially increases with the increase in linker length from 4 to 5 before it decreases upon extending further to 6 and 8 linker atoms. Not surprisingly, the optimum linker length of five corresponds to that found in natural siderophores that contain lysine or di-serine-based linkers, such as enterobactin and azotochelin.

Site-directed mutagenesis of CeuE revealed that Y288 is essential for binding, as the binding affinity observed in the fluorescence titration experiments is far weaker for all mutants containing Y228F when compared to the wild type protein. This finding is confirmed by the lack of CD signal corresponding to the Λ-configuration when Fe-5-Lic is added to these mutants. Mutant H227L retained the capacity to bind ligands, though with reduced affinity compared to wild type, Table 1, suggesting the histidine is not essential to binding. This is supported by the structure of the H227L mutant in complex with Fe-5-Lic, where a water molecule completes the octahedral coordination of Fe(III). Circular dichroism indicated that the Λ-configured ligand arrangement observed in the crystal structure is maintained in solution phase, and that the Λ-configuration is selected by H227L for all Fe-n-Lic species, but with a weaker signal than for wild type CeuE, due to weaker binding affinities across the board.

## Materials and Methods

### Synthesis of 5-LICAM, 6-LICAM and 8-LICAM

The ligands were prepared as previously reported^30^,^31^.

### Cloning, expression and purification

#### Wild type CeuE

All proteins were expressed by standard procedures previously described^20^. The numbering used for *C. jejuni* CeuE starts at the first residue of the mature protein after cleavage of the 20 amino acid signal peptide^20^. The construct 24–310 in our numbering corresponds to residues 44–330 from the Uniprot entry Q0P8Q4_CAMJE for the full length precursor. Four residues (GlyProAlaMet) remain at the N terminus of the construct after 3 C cleavage and the naturally occurring residues start at Leu24.

Protein purification involved standard Ni-NTA-agarose column chromatography to purify His-tagged CeuE. The tag was then cleaved with 3 C protease and dialyzed overnight against 20 mM Tris, pH 7.5, 150 mM NaCl. Final purification involved a second 5 mL Ni-NTA-agarose column with protein elution *via* an imidazole gradient. Non-tagged protein was eluted, concentrated by centrifugal ultrafiltration (Amicon Ultra) and purified by size exclusion chromatography, using a 120 ml Hi load 16/60 Superdex 200, preparative grade column in 20 mM Tris, pH 7.5, 150 mM NaCl. The resulting samples were concentrated to 80 mg mL^−1^.

#### CeuE mutants

PCR-based site directed mutagenesis was used to produce mutants based upon the N-terminal truncated form of CeuE (24–330). The appropriate base mutations were incorporated into amplification primers for plasmid DNA in the YSBLic3C vector. Three single CeuE mutants were constructed: H227L (FWD_H227_L ataaaagtaggcacactcggaaaaagtatcaat; Rev_H227_L attgatactttttccgagtgtgcctacttttat), H227A (FWD_H227_A ataaaagtaggcacagccggaaaaagtatcaat; REV_H227_A attgatactttttccggctgtgcctacttttat) and Y288F (FWD_Y288_F gatccagaatactggtttttagcaagtggaaat; REV_Y288_F atttccacttgctaaaaaccagtattctggatc).

The plasmid DNAs of the CeuE single mutants were then subjected to a second cycle of PCR to yield the double mutants H227L/Y288F and H227A/Y288F. The mutant proteins were expressed and purified according to the standard procedure detailed for wild type CeuE. Pure mutants were concentrated to 20–40 mg/ml and stored at −80 °C.

### Characterization of apo CeuE mutants

Circular dichroism spectroscopy confirmed that all mutants were correctly folded. Electrospray ionization mass spectrometry was used to confirm that all mutations were correctly incorporated. Experimental MWs were within 1 Da of the expected value.

### Crystallization

#### CFe-n-Lic co-crystals

Protocols analogous to those used in the preparation of CFe-4-Lic were followed^20^. For co-crystallization, a solution of CeuE was diluted to 20 mg mL^−1^ in a buffer of 20 mM Tris, 150 mM NaCl at pH 7.5. 114 μL of this solution were mixed with 6 μL of the Fe-5-Lic stock solution to give a 1:10 molar ratio, and 4 mL of protein buffer were added to ensure all the ligand remained in solution. To wash out the excess ligand, additional buffer was added and the diluted solution was re-concentrated using Amicon Centrifugal filter units to a final protein concentration of 20 mg mL^−1^ (measured by following the Bradford method). The solution was centrifuged to remove precipitate, and concentrated to a final volume of ~400 μL. For Fe-6-Lic, 57 μL of CeuE diluted to 20 mg mL^−1^ was mixed with 3 μL of the Fe-6-Lic stock solution to give a 1:10 molar ratio. CFe-8-Lic was prepared in the same way but in a ligand:protein molar ratio of 1:100. H227L-Fe-5-Lic was prepared in a 1:4 molar ratio.

Screening was performed in 96-well plates in a Mosquito nanoliter pipetting robot (TTP LabTech, UK) using sitting-drop vapor diffusion with commercial PACT (Molecular Dimensions) and JCSG screens. Each drop contained 150 nL complex solution and 150 nL reservoir solution. For CFe-5-Lic the best diffraction was observed for crystals grown from two similar PACT conditions, H6 (0.2 M sodium formate, 0.1 M BisTrisPropane, pH 8.5, 20% PEG 3350) and H12 (0.2 M sodium malonate, 0.1 M BisTrisPropane, pH 8.5, 20% PEG 3350). A crystal from condition H12 was used for data collection. For CFe-6-Lic the best diffraction was observed for those grown from two similar PACT conditions, C3 (0.1 M PCB buffer comprising 2:1:2 molar ratio sodium propionate, sodium cacodylate, and BisTrisPropane, pH 6, 25% PEG 1500) and C4 (0.1 M PCB buffer comprising 2:1:2 molar ratio sodium propionate, sodium cacodylate, and BisTrisPropane, pH 7, 25% PEG 1500). A crystal from C4 was cryo-protected (0.1 M PCB buffer comprising 2:1:2 molar ratio sodium propionate, sodium cacodylate, and BisTrisPropane, pH 7, 32.5% PEG 1500) and used for data collection. For CFe-8-Lic the best diffracting crystals grew in PACT conditions, B1 0.1 M MIB buffer pH 4, 25% PEG 1.5 K and H6 0.2 M sodium formate, 0.1 M BisTrisPropane buffer, pH 8.5, 20% PEG 3350. A crystal from H6, cryo-protected in 0.2 M sodium formate, 0.1 M BisTrisPropane buffer, pH 8.5, 32.5% PEG 3350, was used for data collection. For H227L-Fe-5-Lic the best diffracting crystals grew in PACT conditions, 0.1 M PCB buffer, pH 8, 20% PEG 1.5 K.

#### CeuE mutants

Crystals were grown and the structures solved of all generated mutants. Crystals were obtained from the following sitting drop vapor diffusion screening conditions: H227L (0.2 M NaBr; 0.1 M BTP, pH 8.5; 20% PEG 3350); H227A (0.1 M MMT, pH 9.0; 25% PEG 1.5 K) Y288F (0.01 M ZnCl_2_; 0.1 M MES, pH 6.0; 20% PEG 6 K); H227L/Y288F (0.1 M MIB, pH 9.0; 25% PEG 1.5 K); H227A/Y288F (0.1 M SPG, pH 9.0; 25% PEG 1.5 K). All crystals were cryoprotected in the relevant well solution with an increased PEG concentration (20% increased to 36%, 25% increased to 41%) before vitrification at 110 K.

### Structure determination

Data were collected at the Diamond Light Source. Computations were carried out using programs from the CCP4 suite^32^, unless otherwise stated. Diffraction images were processed using *XIA*2^33^,^34^,^35^. The structures were solved with MOLREP^36^ or PHASER^37^, and refined by *REFMAC*^38^ iterated with manual model building/correction in *COOT*^39^. Validation was performed using PROCHECK^40^ and MOLPROBITY^41^.

#### CFe-n-Lic complexes

The CFe-5-Lic crystal was cryo-protected with 35% PEG 3350 and vitrified at 110 K. The CFe-6-Lic and CFe-8-Lic crystals were cryo-protected with 32.5% PEG 1500 and 32.5% PEG 3350 respectively and vitrified at 110 K. H227L-Fe-5-Lic was cryo-protected with 32.5% PEG 1.5 K vitrified at 110 K. The CFe-5-Lic and CFe-6-Lic were isomorphous in space group *P*2_1_2_1_2_1_, with a single protein monomer in the asymmetric unit. The CFe-8-Lic crystal was also in space group *P*2_1_2_1_2_1_, but with different cell dimensions and crystal packing. H227L-Fe-5-Lic was in space group P1. The structures were solved starting with CFe-4-Lic (**PDB ID: 5A1J**) as a search model. Restraints for the ligands were modelled using JLigand^42^. Crystallographic statistics are detailed in Table 2.

#### CeuE mutants

All mutant crystals were cryo-protected with well solution containing 32.5% of relevant PEG and vitrified at 110 K. H227L, H227A, H227L/Y288F and H227A/Y288F were in space group *P*1 with three protein monomers in the asymmetric unit. Y288F was in space group *P*3_2_21 with one protein monomer in the asymmetric unit. Structures were solved starting with apo wild type CeuE (**PDB ID: 3ZKW**) as a search model. The overall protein fold for each mutant was essentially identical to that of wild type CeuE. There was no well-defined electron density for a small number of surface loops, indicating these regions were disordered in the crystal.

There was clear electron density confirming the presence of phenylalanine in place of tyrosine in all three Y288F containing structures. However, H227 is located on a flexible loop and the electron density for the H227A and H227L side chain was absent in these structures. Crystallographic statistics for all mutant structures are detailed in Tables 3 and 4.

### Fluorescence spectroscopy

Fluorescence quenching studies with CeuE were carried out on a Hitachi F-4500 fluorescence spectrophotometer as previously reported^22^.

A 240 nM solution of CeuE in 40 mM Tris-HCl pH 7.5, 150 mM NaCl was prepared and the concentration verified by both the Bradford method and UV-vis absorbance at 280 nm, using the molar absorbance coefficient predicted by ProtParam (15930 mol^−1^ dm^3^ cm^−1^)^43^. The CeuE solution (2 mL) was titrated stepwise with 16 aliquots of a 12 μM Fe-n-Lic solution resulting in a concentration range of Fe-n-Lic of 0 to 1.19 μM. The observed emission intensity was corrected for photomultiplier tube response and buffer subtracted, then integrated between 310 and 410 nm. The data were normalized and analyzed by nonlinear regression using a one-site binding model in DynaFit^26^. Each system was analyzed in triplicate and the average dissociation constants were calculated as reported previously^44^.

### Circular dichroism

Solutions of wild type CeuE and mutant proteins were diluted to 5 × 10^−5^ M in buffer containing 150 mM NaCl and 0.11 M Tris, adjusted to pH 7.5. A Fe(III)-n-Lic (n = 4, 5, 6, 8) solution containing equimolar NTA was prepared at a concentration of 5 × 10^−4^ M.

Spectra were recorded using a solution containing 880 μL 0.11 M Tris pH 7.5, 150 mM NaCl buffer, 100 μL (Fe(III)-n-Lic (n = 4, 5, 6, 8) NTA solution and 20 μL to 5 × 10^−5^ M CeuE, resulting in a 50:1 excess of ligand to ensure full binding.

The spectra were recorded from 300–700 nm and buffer subtracted (data pitch 0.5 mm, scan speed 100 nm min^−1^, response 2 seconds, bandwidth 2 nm, path length 1 cm). The spectra were recorded 5 times and averaged.

### Data availability

Structural data are available in the EMBL-EBI PDB under the accession numbers 5A5D, 5A5V, 5AD1, 5LWQ, 5TCY, 5MBQ, 5LWH, 5MBT and 5MBU. Experimental data were deposited with the University of York library (www.york.ac.uk/library/info-for/researchers/datasets/) under doi: 10.15124/4af725b8-aa8d-4ed5-aa25-48176a795f12.

## Additional Information

**How to cite this article:** Wilde, E. J. *et al*. Interactions of the periplasmic binding protein CeuE with Fe(III) n-LICAM^4−^ siderophore analogues of varied linker length. *Sci. Rep.* **7**, 45941; doi: 10.1038/srep45941 (2017).

**Publisher's note:** Springer Nature remains neutral with regard to jurisdictional claims in published maps and institutional affiliations.

## Figures and Tables

**Figure 1:**
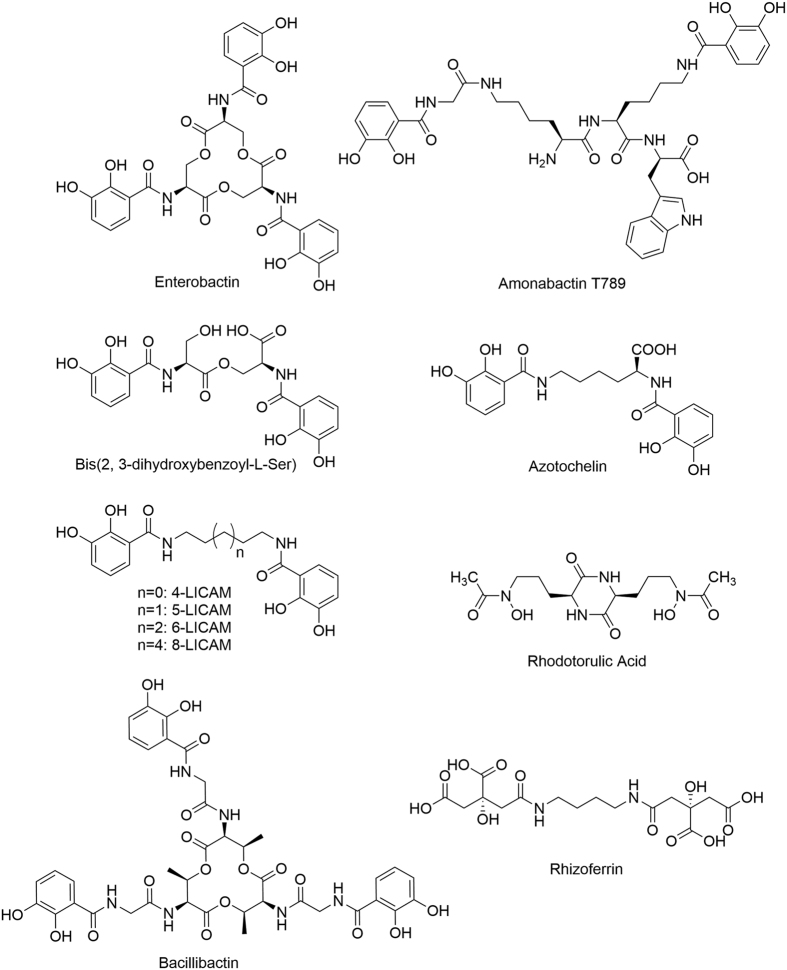
Molecular structures of a representative set of siderophores.

**Table 1:** Dissociation constants.

Fe-n-Lic	WT CeuE K_d_/nM	H227L K_d_/nM	H227A K_d_/nM
4	21 ± 6	90 ± 30	131 ± 28
5	<10	22 ± 10	35 ± 14
6	33 ± 8	65 ± 21	41 ± 7
8	58 ± 8	112 ± 21	125 ± 36

**Table 2:** Crystallographic statistics: wild type Fe-n-Lic complexes.

**Data collection**			
**Structure**	CeuE-[Fe^III^(5-LICAM)]^−^	CeuE-[Fe^III^(6-LICAM)]^−^	CeuE-[Fe^III^(8-LICAM)]^−^
**Beamline**	Diamond 104	Diamond 104	Diamond 104
Wavelength (Å)	0.979	0.979	0.979
**Space group**	P2_1_2_1_2_1_	P2_1_2_1_2_1_	P2_1_2_1_2_1_
**Cell parameters (Å)**	a = 61.52	a = 61.37	a = 42.98
	b = 67.51	b = 66.08	b = 55.98
	c = 69.52	c = 68.96	c = 140.08
**Resolution range (Å)**	46.07–1.74	44.96–2.04	55.98–1.32
**Observations**	190469	146372	457991
**Unique Reflections**	30349	18467	77761
**Monomers in AU**	1	1	1
**Completeness %**^**a**^	99.8 (99.6)	100.0 (100.0)	99.4 (76.0)
I/I (σ)^a^	19.7 (2.7)	9.6 (1.7)	11.0 (1.3)
**CC (1/2)**^**a,b**^	0.999 (0.853)	0.993 (0.583)	0.998 (0.536)
**Average Multiplicity** ^**a**^	6.3 (6.5)	7.9 (8.2)	5.9 (3.1)
**Rmerge (%)**^**a,b,c**^	5.5 (71.9)	20.6 (123.6)	8.3 (85.1)
**Refinement statistics**			
**% R**_**free**_ **reflections**	5	5	5
**%R**_**cryst**_ **(%)**	17.8	21.3	14.0
**Free R factor (%)**	22.8	26.3	18.1
Bond distances (Å)^d^	0.019 (0.019)	0.014 (0.019)	0.020 (0.019)
Bond angles (°)^d^	1.959 (1.994)	1.638 (1.997)	1.960 (1.999)
Chiral centers (Å^3^) ^d^	0.118 (0.200)	0.094 (0.200)	0.133 (0.200)
Planar groups (Å) ^d^	0.010 (0.020)	0.007 (0.021)	0.010 (0.021)
Average B value (Å^2^)	27.7	23.4	13.9
**Main chain (Å**^**2**^)	25.3	23.3	11.0
**Side chain B (Å**^**2**^)	30.1	19.4	14.8
**No. of waters**	266	80	412
**Ramachandran Plot**			
Preferred regions %	89.5	96.4	97.8
Allowed regions %	10.5	3.2	2.2
Outliers %	0	0.4	0
**PDB code**	**5A5D**	**5A5V**	**5AD1**

**Table 3:** Crystallographic statistics: H227 mutant.

**Data collection**			
**Structure**	H227L	H227L-[Fe^III^(5-LICAM)]^−^	H227A
**Beamline**	Diamond 103	Diamond 102	Diamond 103
Wavelength (Å)	0.976	0.979	0.976
**Space group**	P1	P1	P1
**Cell parameters (Å)**	a = 56.92	a = 58.70	a = 56.90
	b = 62.56	b = 62.88	b = 62.61
	c = 67.79	c = 69.87	c = 67.79
**Resolution range (Å)**	65.52–1.52	67.13–1.90	66.02–1.33
**Observations**	631986	254669	598024
**Unique Reflections**	189953	70811	192548
**Monomers in AU**	3	3	3
**Completeness %**^**a**^	95.4 (94.6)	95.9 (96.0)	99.8 (41.0)
I/I (σ)^a^	9.5 (2.3)	6.2 (1.3)	11.8 (1.4)
**CC (1/2)**^**a,b**^	0.996 (0.710)	0.991 (0.418)	0.998 (0.604)
**Average Multiplicity**^**a**^	3.5 (3.5)	3.4 (3.5)	3.4 (3.0)
**Rmerge (%)**^**a,b,c**^	6.0 (48.6)	10.2 (95.6)	4.1 (63.9)
**Refinement statistics**			
**% R**_**free**_ **reflections**	5	5	5
**%R**_**cryst**_ **(%)**	18.5	17.1	14.8
**Free R factor (%)**	22.2	21.5	19.3
Bond distances (Å)^d^	0.024 (0.019)	0.018 (0.019)	0.033 (0.019)
Bond angles (°)^d^	2.338 (1.983)	2.002 (1.850)	2.736 (1.985)
Chiral centers (Å^3^)^d^	0.169 (0.200)	0.114 (0.200)	0.200 (0.200)
Planar groups (Å)^d^	0.011 (0.021)	0.009 (0.021)	0.013 (0.021)
Average B value (Å^2^)	25.7	34.8	25.2
**Main chain (Å**^**2**^)	25.3	32.5	23.4
**Side chain B (Å**^**2**^)	28.0	37.5	27.1
**No. of waters**	301	153	526
**Ramachandran Plot**			
Preferred regions %	96.1	96.3	96.2
Allowed regions %	3.7	3.2	3.7
Outliers %	0.2	0.5	0.1
**PDB code**	**5LWQ**	**5TCY**	**5MBQ**

**Table 4:** Crystallographic statistics: Y288 and double mutants.

**Data collection**			
**Structure**	Y288F	H227L/Y288F	H227A/Y288F
**Beamline**	Diamond 102	Diamond 103	Diamond 103
Wavelength (Å)	0.979	0.979	0.979
**Space group**	P3_2_21	P1	P1
**Cell parameters (Å)**	a = 65.52	a = 56.72	a = 56.90
	b = 65.52	b = 62.36	b = 62.61
	c = 145.65	c = 67.71	c = 67.79
**Resolution range (Å)**	56.74–1.47	60.77–1.80	65.80–1.81
**Observations**	606243	271678	262644
**Unique Reflections**	62546	78620	77003
**Monomers in AU**	1	3	3
**Completeness %**^a^	100.0 (100.0)	96.2 (96.4)	99.6 (96.2)
I/I (σ)^a^	21.7 (1.8)	7.4 (1.2)	8.8 (1.6)
**CC (1/2)**^a^,^b^	1.000 (0.635)	0.995 (0.459)	0.996 (0.599)
**Average Multiplicity**^a^	9.7 (8.9)	3.5 (3.4)	3.4 (3.3)
**Rmerge (%)**^a^,^b^,^c^	4.5 (119.1)	7.6 (81.4)	6.6 (67.5)
**Refinement statistics**			
% R_free_ reflections	5	5	5
%R_cryst_ (%)	12.8	19.4	20.0
Free R factor (%)	16.2	23.9	23.9
Bond distances (Å)^d^	0.030 (0.019)	0.018 (0.019)	0.019 (0.019)
Bond angles (°)^d^	2.456 (1.994)	1.864 (1.983)	1.961 (1.981)
Chiral centers (Å^3^)^d^	0.213 (0.200)	0.117 (0.200)	0.123 (0.200)
Planar groups (Å)^d^	0.006 (0.020)	0.008 (0.021)	0.009 (0.021)
Average B value (Å^2^)	27.6	36.0	35.9
**Main chain (Å**^**2**^)	23.5	34.1	34.7
**Side chain B (Å**^**2**^)	30.0	38.1	37.4
**No. of waters**	186	121	109
**Ramachandran Plot**			
Preferred regions %	96.4	97.1	96.4
Allowed regions %	3.6	2.8	2.9
Outliers%	0	0.1	0.7
**PDB code**	**5LWH**	**5MBT**	**5MBU**

^a^values in parentheses correspond to the highest resolution shell.

^b^CC (1/2) is defined as the Pearson correlation coefficient for two half datasets.

^c^R_merge_ is defined as 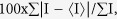 where I is the intensity of the reflection.

^d^R.m.s. deviations from ideal geometry (target values are given in parentheses).
